# Two foreign language automatisms in complex partial seizures^☆^

**DOI:** 10.1016/j.ebcr.2012.10.005

**Published:** 2012-11-08

**Authors:** Hulusi Kececi, Yildiz Degirmenci, Hatice Gumus

**Affiliations:** Duzce University School of Medicine, Neurology Department, Duzce, Turkey

**Keywords:** Temporal lobe epilepsy, Complex partial seizures, Foreign ictal speech automatisms

## Abstract

Ictal speech manifestations with or without loss of consciousness can frequently occur in TLE in which sometimes the patient may remain responsive, even in conjunction with automatisms. Foreign language ictal speech automatism (FLISA) is a rare ictal sign in temporal lobe epilepsy arising from the non-dominant hemisphere. While our literature review revealed no report of CPSs with two foreign language ictal speech automatisms, we here represented a rare case of a Turkish woman with TLE experiencing two foreign language ictal speech automatisms.

## 1 Introduction

Temporal lobe epilepsy (TLE) is a common form of partial epileptic syndromes, mainly observed in adults (70%) [1]. The ictal phenomena of TLE can be divided into broad categories such as motor, sensory, autonomic, experiential, emotional, cognitive, and psychiatric. Temporal lobe epilepsy has a wide profile of ictal symptoms and signs including early ipsilateral-forced contralateral head deviation, contralateral dystonic posturing, behavior and/or mood changes, unilateral hand automatisms, as well as ictal speech automatisms. However, the specificity of these signs is unclear; some of these signs are only of limited value in lateralizing the side of the focus [2], [3], [4].

Ictal speech manifestations with or without loss of consciousness can frequently occur in TLE in which sometimes the patient may remain responsive, even in conjunction with automatisms. Verbal automatisms may be repetitive or non-repetitive but when present, usually occur in the patient's native language, highly suggestive of non-dominant hemisphere involvement [3]. Foreign language ictal speech automatism (FLISA) is a rare ictal sign in temporal lobe epilepsy arising from the non-dominant hemisphere [1].

While our literature review revealed no report of CPSs with two foreign language ictal speech automatisms, we present a rare case of a Turkish woman with TLE experiencing two foreign language ictal speech automatisms.

## 2 Case report

A 19-year-old right-handed Turkish woman presented to our outpatient Neurology clinic with abrupt impairment of consciousness and awareness, altering her ability to interact with her environment. She had motionless stares and abnormal gestures and mimics which were recognized by her teachers during the school time. She was diagnosed at age 5 years with complex partial seizures (CPSs) that occasionally secondarily generalized. She had no significant birth history except for low birth weight. Her generalized seizures were controlled on the combination of oxcarbazepine (OXC), valproic acide (VPA) and levetiracetam (LEV), but her CPSs continued at about 1 to 2 per month, characterized by loss of consciousness, mood changes, déjà vu, and speech automatisms. She had studied English and, to a lesser extent German, in school, without taking a particular interest in either foreign language. Nonetheless, during ictal speech automatisms, while she could not speak or understand Turkish, she could communicate with fluent English and German, though her level of oral comprehension and speech production was limited to simple sentences. Additionally, she couldn't recognize these events during the postictal period.

**Fig. 1:**
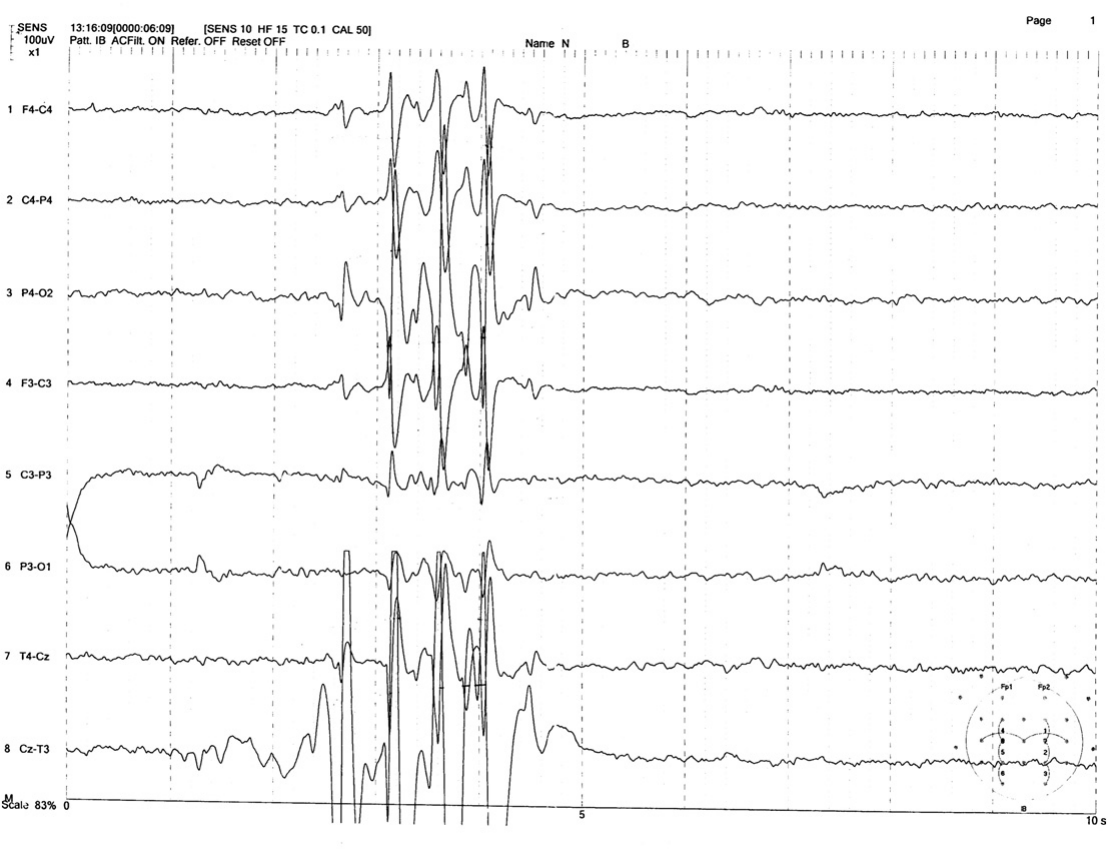
Interictal EEG: generalized spike-wave discharges.

**Fig. 2:**
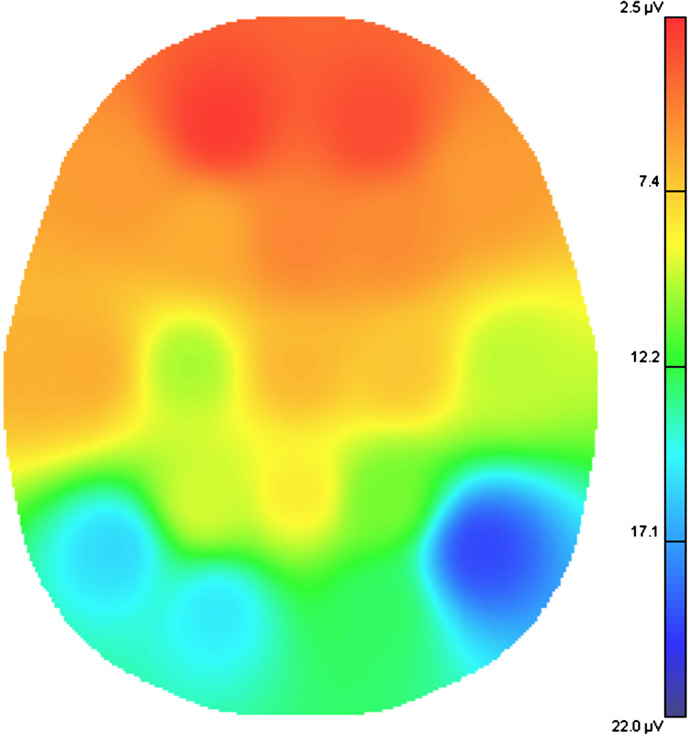
Potential brain mapping of the patient revealing right temporal focus dominancy.

On examination, the patient was alert and oriented. There were no abnormalities on neurological examination. Cranial magnetic resonance imaging (MRI) was also normal. Compatible with the reports of her previous electroencephalography (EEG) recordings, her interictal EEG showed generalized spike–wave discharges (Fig. 1) as well as recurrent bilateral temporal lobe spikes lasting 1–2 s which were dominant over the right hemisphere. No changes were seen with photic stimulation or hyperventilation. Potential brain mapping of the patient revealing right temporal focus dominancy, as shown in Fig. 2.

Her antiepileptic drugs were adjusted to VPA and topiramate with improvement in CPS frequency.

## 3 Discussion

Various forms of language dysfunction have been associated with seizures, especially temporal lobe onset seizures, including loss of speech, dysphasia, and speech automatisms [5], [6], [7].

Ictal speech automatisms are usually uttered in the patient's native language, but rare cases of second language ictal speech production have been reported [5], [6], [7], [8]. Our patient is presented as one of these rare cases with two foreign language ictal automatisms (FLISA). Differing from a previous report suggesting that foreign language ictal speech automatisms are more likely to occur in men [8], we observed this seizure pattern in a teenage girl.

In men or women, native ictal speech automatisms and FLISA are generally accepted to result from partial seizures originating from the non-dominant hemisphere and are related with the amygdala to some extent [6], [7], [8], [9], [10], [11]. There are various reports demonstrating the electroencephalographic projection of these speech automatisms in the temporal lobe of the non-dominant hemisphere. However, they are also noted in the dominant hemisphere [5], [7], [8]. Consistent with previous reports, the ictal EEG in our patient showed bilateral epileptiform activity, predominant on the right. The explanation of this lateralization pattern in ictal speech automatisms with native or foreign language consists of two possible mechanisms according to Driver et al. [5]. They suggested that ictal speech in foreign language results from the release of the dominant hemisphere from the inhibitory action of the non-dominant one, or ictal speech results directly from over activation of the non-dominant hemisphere [5]. These hypotheses were assessed by a previous study using functional MRI and single-photon emission computed tomography, demonstrating that ictal speech automatisms may occur if the foreign language-related widespread network is correctly activated, explaining the rarity of this symptom in comparison with ictal automatisms in native language elicited by a more restricted brain area. These analyses showed that the networks activated by the seizure and those activated by foreign language processing intersected in the right hippocampus [6].

Our patient presented with two foreign language ictal speech automatisms as a rare presentation of TLE. Preservation or activation of both foreign languages during seizures may provide insight to a shared anatomical and neuronal network of foreign languages. Cases such as our patient suggest that the localization of learned foreign languages may be separate from networks supporting native language.
